# Bidirectional Modulation of Alcohol-Associated Memory Reconsolidation through Manipulation of Adrenergic Signaling

**DOI:** 10.1038/npp.2015.248

**Published:** 2015-09-16

**Authors:** Moritz J W Schramm, Barry J Everitt, Amy L Milton

**Affiliations:** 1 Department of Psychology, University of Cambridge, Cambridge, UK

## Abstract

Alcohol addiction is a problem of great societal concern, for which there is scope to improve current treatments. One potential new treatment for alcohol addiction is based on disrupting the reconsolidation of the maladaptive Pavlovian memories that can precipitate relapse to drug-seeking behavior. In alcohol self-administering rats, we investigated the effects of bidirectionally modulating adrenergic signaling on the strength of a Pavlovian cue-alcohol memory, using a behavioral procedure that isolates the specific contribution of one maladaptive Pavlovian memory to relapse, the acquisition of a new alcohol-seeking response for an alcohol-associated conditioned reinforcer. The *β*-adrenergic receptor antagonist propranolol, administered in conjunction with memory reactivation, persistently disrupted the memory that underlies the capacity of a previously alcohol-associated cue to act as a conditioned reinforcer. By contrast, enhancement of adrenergic signaling by administration of the adrenergic prodrug dipivefrin at reactivation increased the strength of the cue-alcohol memory and potentiated alcohol seeking. These data demonstrate the importance of adrenergic signaling in alcohol-associated memory reconsolidation, and suggest a pharmacological target for treatments aiming to prevent relapse through the disruption of maladaptive memories.

## Introduction

Memory reconsolidation—the process by which memories are destabilized following retrieval, and subsequently restabilized to persist in the brain—has received much attention for its potential utility in the development of treatments for neuropsychiatric disorders in which maladaptive emotional memories play a key role (Diergaarde *et al*, 2008; Milton and Everitt, 2010; Milton, 2013; Torregrossa and Taylor, 2013). One such disorder is drug addiction; this chronic relapsing disorder, in which individuals lose control of their drug use, is characterized by a sustained risk of relapse during abstinence (Tiffany, 1990; Anthony *et al*, 1994). Relapse to drug use can be influenced by a number of factors, including a drinking lapse, ie, drug-induced reinstatement (de Wit, 1996; Lê *et al*, 1998), stress (Erb *et al*, 1996; Shaham and Stewart, 1996; Lê *et al*, 1998) and, importantly, for the current work, environmental cues that have previously been associated with drug use in a Pavlovian manner (de Wit and Stewart, 1981). These Pavlovian conditioned stimuli (CSs) that precipitate relapse to drug-seeking behavior activate maladaptive emotional memories and elicit craving and drug-seeking; thus, disrupting their reconsolidation by the administration of an appropriate amnestic agent could be a useful therapeutic strategy.

One such amnestic agent that has received attention is the *β*-adrenergic receptor antagonist propranolol. It has long been known that the adrenergic system influences the consolidation of emotional memories, with an enhancement of signaling at adrenergic receptors increasing memory strength (Sternberg *et al*, 1985; Introini-Collison and McGaugh, 1986; Sternberg *et al*, 1986; Crowe *et al*, 1990; Ferry *et al*, 1999; Hatfield and McGaugh, 1999) and the administration of antagonists at these receptors impairing memory consolidation (Sternberg *et al*, 1985; Sternberg *et al*, 1986; Introini-Collison *et al*, 1992; Hatfield and McGaugh, 1999). These effects, in addition to those on synaptic plasticity *in vivo* (Bliss *et al*, 1983; Dahl *et al*, 1983) and in slice preparations (Stanton and Sarvey, 1985; Ferry *et al*, 1997) led to the adrenergic system being one of the first targets of investigation in the field of memory reconsolidation. Following early demonstrations that antagonism at *β*-adrenergic receptors could disrupt the reconsolidation of memories underlying auditory conditioned fear in rats (Dębiec and LeDoux, 2004), it was demonstrated that *β*-adrenergic receptor antagonism with propranolol also disrupted the reconsolidation of drug-associated memories in cocaine-conditioned (Fricks-Gleason and Marshall, 2008) and morphine-conditioned (Robinson and Franklin, 2007; Robinson and Franklin, 2010) place preference procedures, and in procedures investigating the capacity of previously cocaine-associated cues to elicit and maintain drug seeking (Milton *et al*, 2008). However, these effects have not been universally replicated, as the memories underlying place preference in chronically morphine-treated rats appear insensitive to propranolol at reactivation (Robinson *et al*, 2011).

The reconsolidation of alcohol-associated memories has been less well-studied than for other drugs of abuse, despite alcohol abuse and addiction presenting a significant societal burden (UK Home Office, 2012). Memories relevant to relapse in alcohol addiction do appear to undergo reconsolidation (von der Goltz *et al*, 2009; Wouda *et al*, 2010; Barak *et al*, 2013), but there have been inconsistent reports in the literature regarding the dependence of this process on *β*-adrenergic signaling. Those few studies that have investigated the effects of propranolol on alcohol-associated memories have shown that propranolol does not disrupt the reconsolidation of the memories underlying alcohol-conditioned place preference (Font and Cunningham, 2012), but does disrupt the reconsolidation of Pavlovian memories that contribute to cue-induced relapse to alcohol seeking (Wouda *et al*, 2010). As cue-induced relapse can be behaviorally deconstructed into the constituent processes of Pavlovian conditioned approach, conditioned motivation, and conditioned reinforcement (the ‘three routes to relapse'—see Milton and Everitt, 2010), this latter report appears to be inconsistent with the finding that propranolol does not disrupt the reconsolidation of the memories underlying Pavlovian conditioned approach or conditioned motivation for alcohol-associated cues (Milton *et al*, 2012). However, this may reflect differential dependence of the reconsolidation of the memories underlying these processes on *β*-adrenergic signaling. Propranolol has also been shown ineffective at disrupting the reconsolidation of the memories underlying Pavlovian conditioned approach and conditioned motivation for sucrose-associated cues (Lee and Everitt, 2008), yet it impairs the reconsolidation of the memory allowing a sucrose-associated cue to act as a conditioned reinforcer (Milton *et al*, 2008). It has also been shown previously that manipulations that fail to impair the reconsolidation of the memories underlying conditioned place preference may disrupt the memories that underlie conditioned reinforcement (Théberge *et al*, 2010). Thus, a key question is whether *β*-adrenergic signaling is required for the reconsolidation of the Pavlovian memory underlying the capacity of a previously alcohol-associated cue to act as a conditioned reinforcer; if it is, then this may provide an explanation for why propranolol disrupts the reconsolidation of the memories underlying some, but not all, measures of relapse to alcohol seeking.

Therefore, in this study, we investigated the effects of adrenergic manipulations on the reconsolidation of an alcohol-associated cue memory and its capacity to act as a conditioned reinforcer. We hypothesized that adrenergic transmission within the brain would be required for the restabilization of a cue-alcohol memory, and its ability to act subsequently as a conditioned reinforcer. We tested this hypothesis using the acquisition of a new instrumental seeking response for conditioned reinforcement (ANR), and by comparing the effects of administering at reactivation the lipophilic *β*-adrenergic receptor antagonist propranolol *vs* the hydrophilic (lipophobic) *β*-adrenergic receptor antagonist nadolol, which does not cross the blood–brain barrier. We further hypothesized that an enhancement of adrenergic signaling would enhance the reconsolidation of an alcohol-associated cue memory, thereby potentiating its impact on alcohol seeking; we tested this hypothesis in a separate experiment by administering the adrenergic prodrug dipivefrin (Kaback *et al*, 1976) before memory reactivation.

## Materials and methods

### Subjects

Subjects were 113 experimentally naive male Lister-Hooded rats (Charles River, Bicester, UK) housed in pairs in a vivarium on a reversed light–dark cycle (lights on at 19:00) and weighing between 201–337 g at the start of the experiments. Subjects were food restricted, although not deprived, and maintained at least 90% of their free-feeding weight. Animals were fed after training or testing each day. Access to water was *ad libitum* except for when inside the conditioning chambers, and for the first 2 days of saccharin fading, when animals were water restricted (without food restriction) for 22 h per day. All procedures were conducted in accordance with the UK Animals (Scientific Procedures) Act 1986.

### Habituation to Ethanol Drinking (Saccharin-Fading Procedure)

Rats were habituated to ethanol drinking using a modified version of the sucrose fading procedure, as described previously (Milton *et al*, 2012). Briefly, they were placed in individual cages containing a single bottle, and were given 1-h long access sessions daily for a total of 14 days. The fluid contained in the bottle varied across sessions: four sessions of 0.2% saccharin, followed by two sessions of 0.2% saccharin+5% ethanol, then two sessions of 5% ethanol, two sessions of 0.2% saccharin+8% ethanol, two sessions of 8% ethanol, and finally two sessions of 0.2% saccharin+10% ethanol (Supplementary Figure S1). Fluid intake in milliliters was estimated by weighing the bottles before and after each session. Following habituation to ethanol drinking, all animals progressed on to ethanol self-administration training.

### Behavioral Procedures

**Figure 1:**
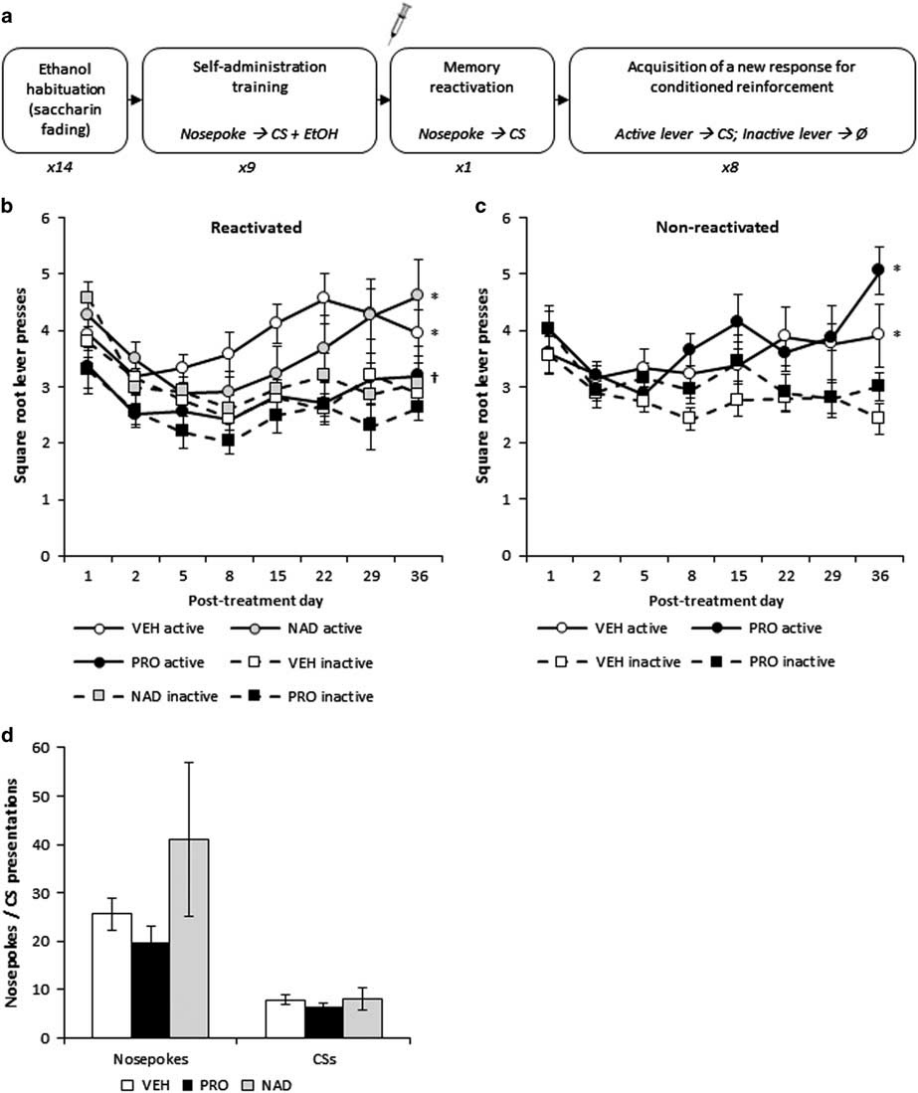
A centrally active, but not peripherally active, *β*-adrenergic receptor antagonist disrupted the reconsolidation of a CS-alcohol memory. (a) An overview of the experimental timeline. The injection symbol represents an i.p. injection of the centrally active *β*-adrenergic receptor antagonist propranolol (PRO), the peripherally active *β*-adrenergic receptor antagonist nadolol (NAD) or vehicle (VEH) 30 min before the memory reactivation session. The numbers underneath the boxes refer to the number of sessions. (b) Administration of PRO before memory reactivation prevented a previously alcohol-associated CS from subsequently acting as a conditioned reinforcer. By contrast, administration of NAD at reactivation did not affect the CS-alcohol memory, such that the CS could subsequently act as a conditioned reinforcer in the same manner that it did for animals treated with VEH at reactivation. Asterisks denote statistically higher responding on the active than inactive lever; the dagger denotes active lever pressing that is significantly lower than the VEH control group. (c) When PRO was administered without a memory reactivation session, it did not subsequently impair the capacity of the alcohol-associated CS to act as a conditioned reinforcer, indicating that the disruption of the CS-alcohol memory with propranolol was reactivation-dependent. Asterisks denote statistically higher responding on the active than inactive lever. Data in b and c are square-root transformed and presented as means±SEM. (d) The administration of *β*-adrenergic receptor antagonists had no acute effect on performance during the memory reactivation session, as PRO nor NAD had any effect on the number of nosepokes made during the memory reactivation, or the number of response-contingent CS presentations earned during this session. Group sizes: (b and d) reactivated VEH=22; reactivated PRO=11; reactivated NAD=9; (c) non-reactivated VEH=13; non-reactivated PRO=13 rats per group.

**Figure 2:**
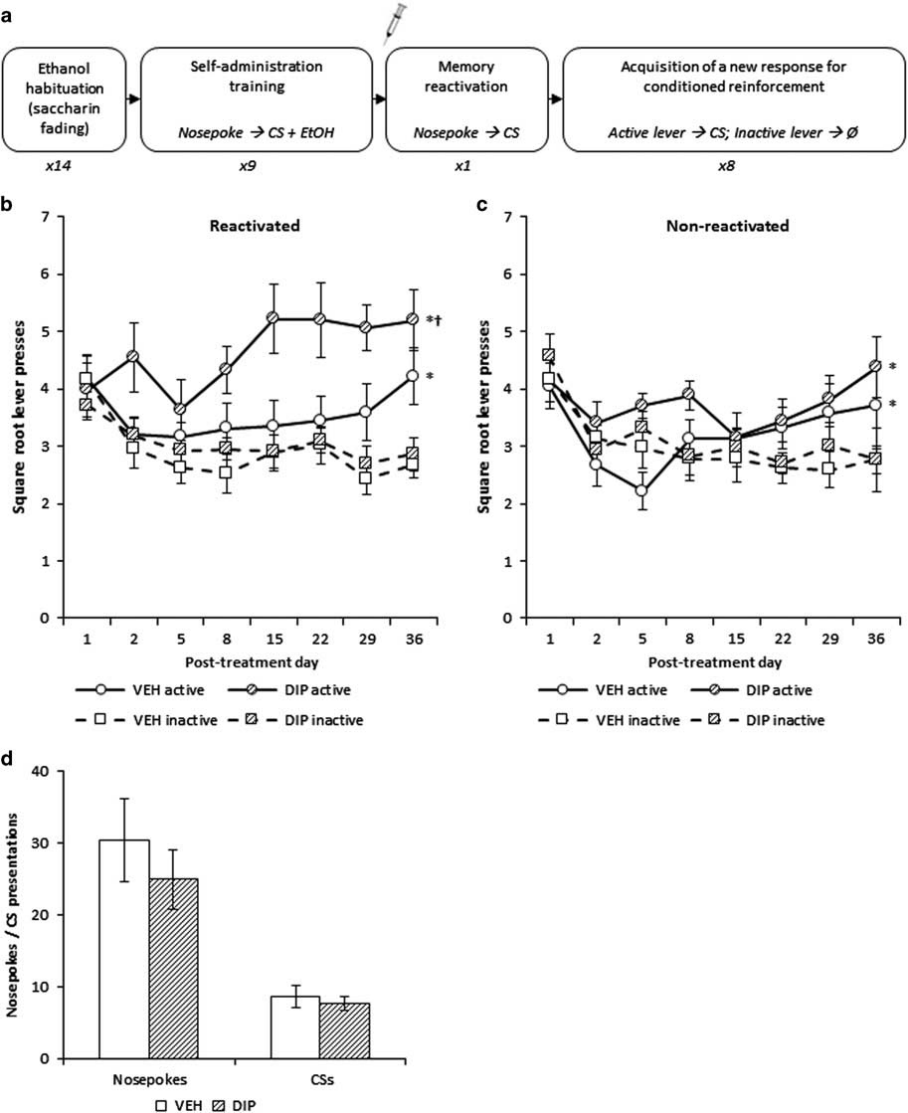
Administration of the adrenergic prodrug dipivefrin (DIP) enhanced the reconsolidation of CS-alcohol memory, increasing subsequent responding for a previously alcohol-associated conditioned reinforcer. (a) An overview of the experimental timeline. The injection symbol represents an i.p. injection of the adrenergic prodrug DIP 10 min before the memory reactivation session. The numbers underneath the boxes refer to the number of sessions. (b) Administration of DIP before the memory reactivation session enhanced subsequent responding on the lever reinforced by presentation of the previously alcohol-associated conditioned reinforcer. Asterisks denote statistically higher responding on the active than inactive lever; the dagger denotes active lever pressing that is significantly higher than the VEH control group. (c) DIP given without memory reactivation produced subsequent responding that was indistinguishable from vehicle-treated animals. Asterisks denote statistically higher responding on the active than inactive lever. Data in b and c are square-root transformed and presented as means±SEM. (d) DIP had no acute effect on performance during the memory reactivation session, as it did not affect the number of nosepoke responses made, or CS presentations earned, relative to the vehicle-treated (VEH) control group during the memory reactivation session. Group sizes: (b and d) reactivated VEH=13; reactivated DIP=14; (c) non-reactivated VEH=9; non-reactivated DIP=9 rats per group.

In order to assess the integrity of the CS-ethanol memory following our experimental manipulations at reactivation, we used the acquisition of a new instrumental response (ANR) procedure (as used to investigate the reconsolidation of cocaine- and sucrose-associated memories in Milton *et al*, 2008) to assess whether the previously ethanol-associated CS was able subsequently to act as a conditioned reinforcer (Figures 1a and 2a). Briefly, in the first phase of the experiment animals were trained to self-administer ethanol with an instrumental response (a nosepoke), which led to the simultaneous presentation of a light, the pavlovian CS. Following the experimental manipulations of memory at reactivation, animals were tested repeatedly in the second phase of the experiment for the acquisition of a novel instrumental response (lever pressing) for the presentation of the CS alone. Pavlovian CSs that are capable of acting as conditioned reinforcers, because of their previous association with the ethanol reinforcer, support the acquisition of a new instrumental response, while CSs that are not (or are no longer) associated with primary reinforcement cannot. Thus, the ANR procedure provides a stringent assay for a specific psychological process that can only be supported when the CS-ethanol memory is intact.

All behavioral procedures were conducted during the animals' dark cycle. Rats were trained in conditioning chambers (Med Associates Inc., St Albans, Vermont) to make a nosepoke response into a central magazine for presentation of a 0.1 ml of a 10% (v/v) ethanol reinforcer (Sigma-Aldrich, Gillingham, UK), which was associated with a 20-s light CS (presented on the same side assigned to the ‘inactive' lever during testing, counterbalanced across rats) on a fixed ratio (FR) 1 schedule. Rats were trained over nine sessions, with the session terminating after 60 min or a maximum of 30 CS-ethanol pairings per session, whichever occurred first.

The day after the completion of training, rats received systemic injections of drugs targeting the adrenergic system 30 min before the memory reactivation session. During this session, nosepokes led to the presentation of the light CS and activation of the pump that delivered ethanol during training on an FR1 schedule, but no ethanol was delivered. The primary reinforcer was omitted at reactivation because it has been hypothesized (Pedreira *et al*, 2004) and is becoming increasingly supported (Forcato *et al*, 2009; Sevenster *et al*, 2013) that memory destabilization processes are initiated by a ‘mismatch' between what is expected by the individual and what occurs during the reactivation session. For this reason, the session also terminated ‘early', after 15 min. During this time, the rats were limited to a maximum of 30 CS presentations, but this limit was not usually reached (see Figures 1d and 2d for the number of CS presentations achieved).

Testing began 24 h after the memory reactivation session. The rats were returned to the same conditioning chambers, but in this phase they were presented with two novel levers (left and right of the central magazine). Depression of the ‘active' lever led to an abbreviated (1 s) presentation of the light CS on a variable ratio schedule (VR1-3), while depression of the ‘inactive' lever had no programmed consequence and acted as a control for general activity. The light CS was always presented on the side opposite to the ‘active' lever, to avoid Pavlovian conditioned approach contributing to lever pressing. No ethanol was available during these sessions. Rats were returned to the chambers for eight 30-min sessions, conducted 1, 2, 5, and 8 days following memory reactivation, and then weekly following day 8 (on days 15, 22, 29, and 36). Lever presses and nosepokes were recorded by computer.

### Systemic Drug Administration

#### Experiment 1—the effects of centrally active *vs* peripherally active *β*-adrenergic receptor antagonists on the reconsolidation of a CS-alcohol memory

All rats received intraperitoneal (i.p.) injections of the lipophilic *β*-adrenergic receptor antagonist propranolol (PRO, 10 mg/kg; Sigma-Aldrich), the hydrophilic *β*-adrenergic receptor antagonist nadolol (NAD, 20 mg/kg; Sigma-Aldrich), or saline vehicle (VEH). Animals that underwent memory reactivation were injected i.p. in a novel room and returned to the home cage for 30 min before the memory reactivation session; non-reactivated control groups received injections in the novel room and were not re-exposed to the conditioning chambers. The dose of propranolol used has been shown previously to disrupt the reconsolidation of Pavlovian CS fear (Dębiec and LeDoux, 2004) and Pavlovian CS-sucrose (Milton *et al*, 2008) memories. Although nadolol is reported to have higher efficacy at *β*-adrenergic receptors than propranolol (Escoubet *et al*, 1986) a higher dose of nadolol was used here to ensure that a behaviorally effective dose was used, and to be consistent with previous literature (Robinson and Franklin, 2007).

#### Experiment 2—the effects of the adrenergic prodrug dipivefrin on the reconsolidation of a CS-alcohol memory

Animals received i.p. injections of either the adrenergic prodrug dipivefrin (DIP) hydrochloride (10 μg/kg, US Pharmacopeial Convention, Rockville, MD, USA) or its saline vehicle (VEH) 10 min before the memory reactivation session. This dose of DIP has been shown to enhance the consolidation of inhibitory avoidance memory when administered immediately after training (Introini-Collison *et al*, 1992). Non-reactivated animals received the same dose of DIP but were returned to the home cage after the injection.

### Sample Size, Statistical Power, and Randomization

*A priori* sample size calculations were not conducted but the number of subjects per group was chosen by reference to previous research. Data were collected over an extended period of time, with 6–8 animals being run within a single squad. Data from different squads were pooled for analysis, with final numbers per group of: (experiment 1) reactivated VEH=22, reactivated PRO=11, reactivated NAD=9, non-reactivated VEH=13, and non-reactivated PRO=13; (experiment 2) reactivated VEH=13, reactivated DIP=14, non-reactivated VEH=9, and non-reactivated DIP=9. Subjects were pseudorandomly assigned to experimental groups, such that drug assignments were made according to training performance (ie, groups were matched for nosepoking performance and the numbers of CSs and ethanol reinforcers earned during training).

### Data Collection and Statistical Analysis

Data were recorded automatically by the Conditioned Reinforcement program (Cardinal, 2005) running within the Whisker Control server (Cardinal, 2000). As the data were collected by computer, blinding to experimental group was not required.

Training and testing data were analyzed using repeated measures ANOVA, and reactivation data were analyzed using a one-way ANOVA. The normality assumption of ANOVA was checked with the Shapiro–Wilk test, and if this indicated that the data were not normally distributed, then they were transformed. The lever pressing and nosepoke data from the ANR phase of the experiment were not normally distributed, so were transformed using the Box–Cox method with *λ*=0.5; ie, square-root transformed. Following this transformation, the majority of the lever press data satisfied the assumption of normality (*p*>0.05).

If Mauchly's test indicated that the assumption of sphericity had been violated, then the Greenhouse–Geisser correction was applied where *ɛ*<0.75, and the Huynh–Feldt correction applied where *ɛ*>0.75, as recommended by Cardinal and Aitken (2006). The *α*-level was 0.05 for all analyses, and *p*-values are two-tailed. Where appropriate, subsequent ANOVAs and Šidák-corrected pairwise comparisons were conducted to investigate specific *a priori* hypotheses.

## Results

### Experiment 1

#### Administration of the lipophilic *β*-adrenergic receptor antagonist propranolol, but not the hydrophilic *β*-adrenergic receptor antagonist nadolol, before memory reactivation disrupted the CS-alcohol memory that subsequently supports conditioned reinforcement

Administration of the lipophilic *β*-adrenergic receptor antagonist propranolol before memory reactivation impaired the capacity of a previously alcohol-associated CS to act as a conditioned reinforcer, in a reactivation-dependent manner (Figure 1b; drug × reactivation: F_(1,55)_=5.46, *p*=0.023, *η*^2^=0.09). Animals that received VEH readily acquired the new instrumental response, increasing responding on the active lever across the eight test sessions (lever: F_(1,33)_=17.9, *p*<0.001, *η*^2^=0.35; lever × session: F_(5.4,180)_=4.99, *p*<0.001, *η*^2^=0.13) with no differences in performance between reactivated (Figure 1b) and non-reactivated (Figure 1c) groups (reactivation: F<1; lever × reactivation: F<1; lever × session × reactivation: F_(5.4,180)_=1.21, *p*=0.30). By contrast, animals that received PRO before reactivation responded less than animals receiving VEH before reactivation (drug: F_(1,31)_=5.45, *p*=0.026, *η*^2^=0.15) and responded less than animals that received PRO without reactivation (reactivation: F_(1,22)_=9.10, *p*=0.006, *η*^2^=0.29). While non-reactivated, PRO-treated animals responded more on the CS-producing active lever over the course of testing (lever: F_(1,12)_=6.79, *p*=0.023, *η*^2^=0.36; lever × session: F_(3.1,37)_=5.42, *p*=0.003, *η*^2^=0.31), animals given PRO before reactivation did not bias their responding towards the active lever over the course of testing (lever: F_(1,10)_=2.17, *p*=0.17; lever × session: F<1). Thus, systemic propranolol, which readily crosses the blood–brain barrier, disrupted the reconsolidation of a CS-alcohol memory that subsequently allowed the CS to act as a conditioned reinforcer.

By contrast, administration of the hydrophilic (lipophobic) *β*-adrenergic receptor antagonist nadolol, which does not cross the blood–brain barrier, at reactivation did not affect the capacity of the CS to act subsequently as a conditioned reinforcer (Figure 1b), as the reactivated NAD group did not differ in performance from the reactivated VEH group (drug: F<1; lever × drug: F<1). Therefore, *β*-adrenergic receptor antagonism only disrupted the reconsolidation of the CS-alcohol memory if it was administered in conjunction with a memory reactivation session, and if the antagonist was centrally active.

The number of nosepoke responses, which had previously been reinforced with alcohol, did not differ between VEH and PRO groups during ANR testing, as has been observed previously for CS-sucrose and CS-cocaine memories (Supplementary Figure S2). There were no differences between the VEH and PRO groups, regardless of whether drug administration had occurred with memory reactivation or not (drug: F<1; reactivation: F_(1,55)_=3.45, *p*=0.069; drug × reactivation: F<1). Interestingly, rats that received NAD before memory reactivation made more nosepokes during the ANR test sessions than the VEH group (drug: F_(1,29)_=5.47, *p*=0.026, *η*^2^=0.16; session × drug: F_(4.0,117)_=3.97, *p*=0.005, *η*^2^=0.12]. Šidák-corrected pairwise comparisons revealed higher numbers of nosepokes made by the NAD group in the first, second, and fourth ANR test sessions (all *p*'s<0.012) but this increased nosepoking was not persistent, and returned to VEH levels from the fifth test session (all *p*'s>0.16).

#### Neither propranolol nor nadolol acutely affected performance during the memory reactivation session

Neither of the *β*-adrenergic receptor antagonists acutely affected responding during the memory reactivation session (Figure 1d). The number of CS presentations was the same in all reactivated groups (drug: F<1) and there were no differences in the numbers of nosepokes made during the memory reactivation session (drug: F_(2,39)_=1.86, *p*=0.17).

#### All experimental groups were matched for acquisition of the CS-alcohol association during training

There were no differences between groups in the acquisition of alcohol-drinking behavior as assessed by ethanol consumption during the saccharin-fading procedure (Supplementary Figure S1), and no differences in the acquisition of the CS-alcohol memory during training (Supplementary Figure S3). Rats subsequently given either VEH or PRO on the treatment day did not differ in terms of the amount of ethanol drunk during the saccharin-fading procedure (drug: F<1), and there were no differences in ethanol consumption between prospective reactivated and non-reactivated groups (reactivation: F_(1,55)_=3.85, *p*=0.055). There were no differences in the amount of fluid consumed between the prospective reactivated VEH group and the prospective NAD group (drug: F_(1,29)_=1.24, *p*=0.28; session × drug: F<1).

The prospective VEH and PRO groups were well-matched for performance during nosepoke training (Supplementary Figure S3). All rats acquired the instrumental nosepoke response for ethanol across the course of training (session: F_(4.6,251)_=3.02, *p*=0.014, *η*^2^=0.05) and there were no differences in the number of nosepokes made by the prospective VEH- or PRO-treated rats (drug: F<1) nor any differences between those rats that were subsequently reactivated and those that were not (reactivation: F<1). There were also no differences in the number of nosepokes made during training between the prospective reactivated VEH group and the prospective NAD group (drug: F<1).

Similarly, there were no differences in the number of CS-alcohol pairings during training. The number of behaviorally contingent CS exposures increased across sessions (session: F_(4.6,255)_=8.16, *p*<0.001, *η*^2^=0.13) and there were no differences in the number of nosepokes made by the prospective VEH- or PRO-treated rats (drug: F*<*1) nor any differences between those rats that were subsequently reactivated and those that were not (reactivation: F_(1,55)_=2.26, *p*=0.14). There were also no differences in the number of CS exposures during training between the prospective VEH and NAD groups (drug: F_(1,29)_=2.28, *p*=0.14).

### Experiment 2

#### Administration of the adrenergic prodrug dipivefrin at reactivation enhanced the capacity of a previously alcohol-associated CS to act subsequently as a conditioned reinforcer

Administration of the adrenergic prodrug DIP before memory reactivation enhanced the capacity of the previously alcohol-associated CS to support the acquisition of a new instrumental response for conditioned reinforcement in subsequent test sessions (drug: F_(1,41)_=4.36, *p*=0.043, *η*^2^=0.096). All animals acquired the new instrumental response for conditioned reinforcement (Figure 2b and c), discriminating between the CS-producing active lever and the inactive control lever (lever: F_(1,41)_=20.1, *p*<0.001, *η*^2^=0.33), with a trend towards slightly better discrimination by the DIP-treated group (lever × drug: F_(1,41)_=3.59, *p*=0.065, *η*^2^=0.08) and by reactivated animals (lever × reactivation: F_(1,41)_=4.06, *p*=0.051, *η*^2^=0.09). Although there was no significant interaction of lever × reactivation × drug (F<1), this comparison was somewhat underpowered, with an observed power of 0.14. However, *a priori* planned Šidák-corrected pairwise comparisons revealed that animals that received DIP before reactivation responded more on the active lever than VEH-treated controls (*p*=0.017), while there were no differences between the non-reactivated DIP- and VEH-treated groups (*p*=0.339). Furthermore, there were no differences in responding on the inactive lever for any of the groups (all *p*'s>0.62).

There were no differences in the number of nosepokes made during ANR testing (Supplementary Figure S2) by animals treated with DIP or VEH, regardless of whether the drugs were administered before, or in the absence of, memory reactivation (drug: F<1; reactivation: F<1; drug × reactivation: F<1).

#### Administration of dipivefrin before memory reactivation did not acutely affect behavior during the reactivation session itself

Responding during the memory reactivation session was unaffected by the prior administration of DIP (Figure 2d). There were no differences in the numbers of nosepokes made by the two experimental groups (drug: F<1) and consequently no differences in the number of response-contingent CS presentations during reactivation (drug: F<1).

#### All experimental groups were matched for acquisition of the CS-alcohol association during training

There were no differences in the amount of ethanol consumed by the different experimental groups during the saccharin-fading procedure (Supplementary Figure S1). Rats subsequently assigned to the VEH and DIP groups did not differ on the amount of ethanol drunk during fading (drug: F<1) and there were no differences between the prospective reactivated and non-reactivated groups (reactivation: F_(1,41)_=2.60, *p*=0.12).

There were no differences between the prospective experimental groups during nosepoke training (Supplementary Figure S3) of the CS-alcohol association (drug: F_(1,41)_=1.12, *p*=0.30; session × drug: F_(5.1,209)_=1.07, *p*=0.39). Although there was a trend towards greater responding in the non-reactivated groups (reactivation: F_(1,41)_=4.01, *p*=0.052, *η*^2^=0.09), this was the case for both the DIP- and VEH-treated groups (drug × reactivation: F<1; session × drug × reactivation: F<1). Likewise, there were no differences in the numbers of CS presentations earned during training between the experimental groups, regardless of whether the groups were to be subsequently reactivated or not (drug: F<1; reactivation: F_(1,41)_=1.52, *p*=0.23; drug × reactivation: F<1).

## Discussion

The experiments described here demonstrate that enhancing and diminishing activity of central *β*-adrenergic receptors can bidirectionally modulate the reconsolidation of a CS-alcohol memory, as assessed by the capacity of the previously alcohol-associated CS to act as a conditioned reinforcer in rats with a history of alcohol self-administration. As has been observed previously for cocaine-, heroin- and sucrose-associated stimuli (Di Ciano and Everitt, 2004; Parkinson *et al*, 2005) responding for conditioned reinforcement was persistent and resistant to extinction in vehicle-treated animals. However, the lipophilic *β*-adrenergic receptor antagonist propranolol, but not the hydrophilic *β*-adrenergic receptor antagonist nadolol, disrupted the reconsolidation of the CS-alcohol memory when administered at memory reactivation. Furthermore, systemic administration of the adrenergic prodrug DIP at reactivation enhanced the reconsolidation of the CS-alcohol memory, increasing the capacity of the previously alcohol-associated CS to act subsequently as a conditioned reinforcer. Thus, these data indicate that the reconsolidation of a CS-alcohol memory can be bidirectionally modulated by reducing and enhancing central adrenergic signaling.

These data extend previous work showing that adrenergic signaling is required for the reconsolidation of the memory underlying conditioned reinforcement for cocaine-associated and sucrose-associated CSs (Milton *et al*, 2008) through our use of alcohol as the primary reinforcer. Along with the demonstration that propranolol can disrupt the reconsolidation of memories underlying a place preference conditioned to morphine (Robinson and Franklin, 2007) or to alcohol (Wouda *et al*, 2010), this work shows that adrenergic signaling is not only required for the reconsolidation of memories associated with psychostimulants, but also for drugs with CNS-depressant mechanisms of action. Furthermore, the demonstration that nadolol failed to disrupt the reconsolidation of the CS-alcohol memory adds to growing evidence that central adrenergic signaling is required for memory reconsolidation; the systemic administration of nadolol also failed to disrupt the reconsolidation of the memory underlying morphine-conditioned place preference (CPP) (Robinson and Franklin, 2007), although nadolol administered directly into the basolateral amygdala did disrupt the reconsolidation of the memory underlying cocaine CPP (Otis *et al*, 2013).

In contrast to previous research, we found that a single treatment of propranolol given at memory reactivation was sufficient to persistently disrupt the CS-US memory for at least 36 days after treatment, whereas multiple reactivation and treatment sessions were required to disrupt the reconsolidation of the memory underlying alcohol CPP (Wouda *et al*, 2010). We have previously shown that propranolol does not disrupt the reconsolidation of the memories underlying alcohol-CS conditioned approach (‘sign-tracking') or conditioned motivation (PIT) (Milton *et al*, 2012). These findings therefore support our hypothesis that propranolol disrupts only one of the ‘three routes to relapse'—that by which drug-associated CSs reinforce drug-seeking responses (Milton and Everitt, 2010). It has been shown previously that conditioned approach, conditioned motivation and conditioned reinforcement are supported by different components of the limbic corticostriatal circuitry (see Cardinal *et al* (2002) for review). Further work will be required to determine whether the capacity of *β*-adrenergic signaling to influence memory reconsolidation is reflected by differences in adrenergic projections and receptor distributions in these areas.

The finding that the reconsolidation of a CS-alcohol memory can be enhanced by the administration of the adrenergic prodrug DIP is the first demonstration, to our knowledge, that an appetitive memory has been strengthened by increasing adrenergic signaling at reactivation. Thus, these data extend previous findings for aversive memories, where the administration of the *β*-adrenergic receptor agonist isoproterenol (Dębiec *et al*, 2011) or administration of the *α*_2_-adrenergic receptor antagonist yohimbine (Gazarini *et al*, 2013) were shown to enhance the reconsolidation of a CS-fear memory.

Disrupting the memories underlying the ‘three routes to relapse' (Milton and Everitt, 2010) offers an opportunity for improving current treatments to promote abstinence in drug addiction. Of these ‘routes to relapse', conditioned reinforcement is particularly problematic because of its persistence and resistance to extinction (Di Ciano and Everitt, 2004). Thus, propranolol may be useful in disrupting the memories that underlie this process in the maladaptive context of addiction. Further work needs to be conducted to determine whether propranolol would be effective in populations with alcohol dependence and whether these results would generalize to previously alcohol-dependent humans, but the finding that a drug that can be safely administered systemically in humans is effective at persistently reducing the risk of relapse in a rodent model of cue-induced relapse holds promise for the development of pro-abstinence, anti-relapse treatments for addiction.

## FUNDING AND DISCLOSURE

This work was supported by a UK Medical Research Council Programme Grant (G1002231) to BJE and ALM and was conducted in the Behavioural and Clinical Neuroscience Institute, an initiative jointly funded by the MRC and the Wellcome Trust. MJWS was supported by an MRC Doctoral Training Grant and the James Baird Fund at the Medical School of the University of Cambridge. ALM was partly supported by a BCNI lectureship and the Ferreras-Willetts Fellowship from Downing College, Cambridge.
