# Supplementary figures and images for: Bidirectional Modulation of Alcohol-Associated Memory Reconsolidation through Manipulation of Adrenergic Signaling
Source: Neuropsychopharmacology. 2015 Sep 16;41(4):1103–11. doi: 10.1038/npp.2015.248 (PMC4663661; doi:10.1038/npp.2015.248)

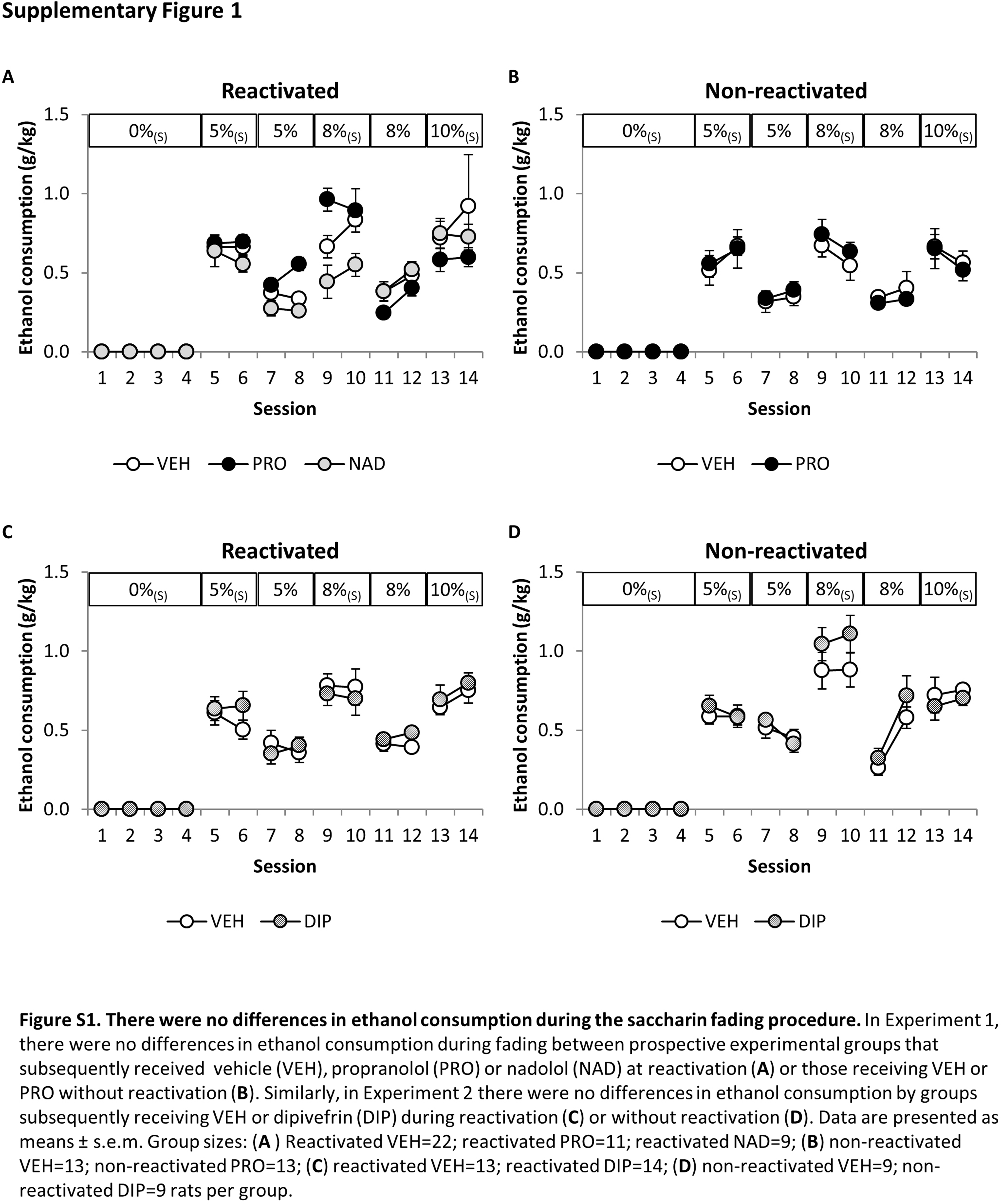

Supplement: Supplementary Figure S1 [file npp2015248x1.tif]

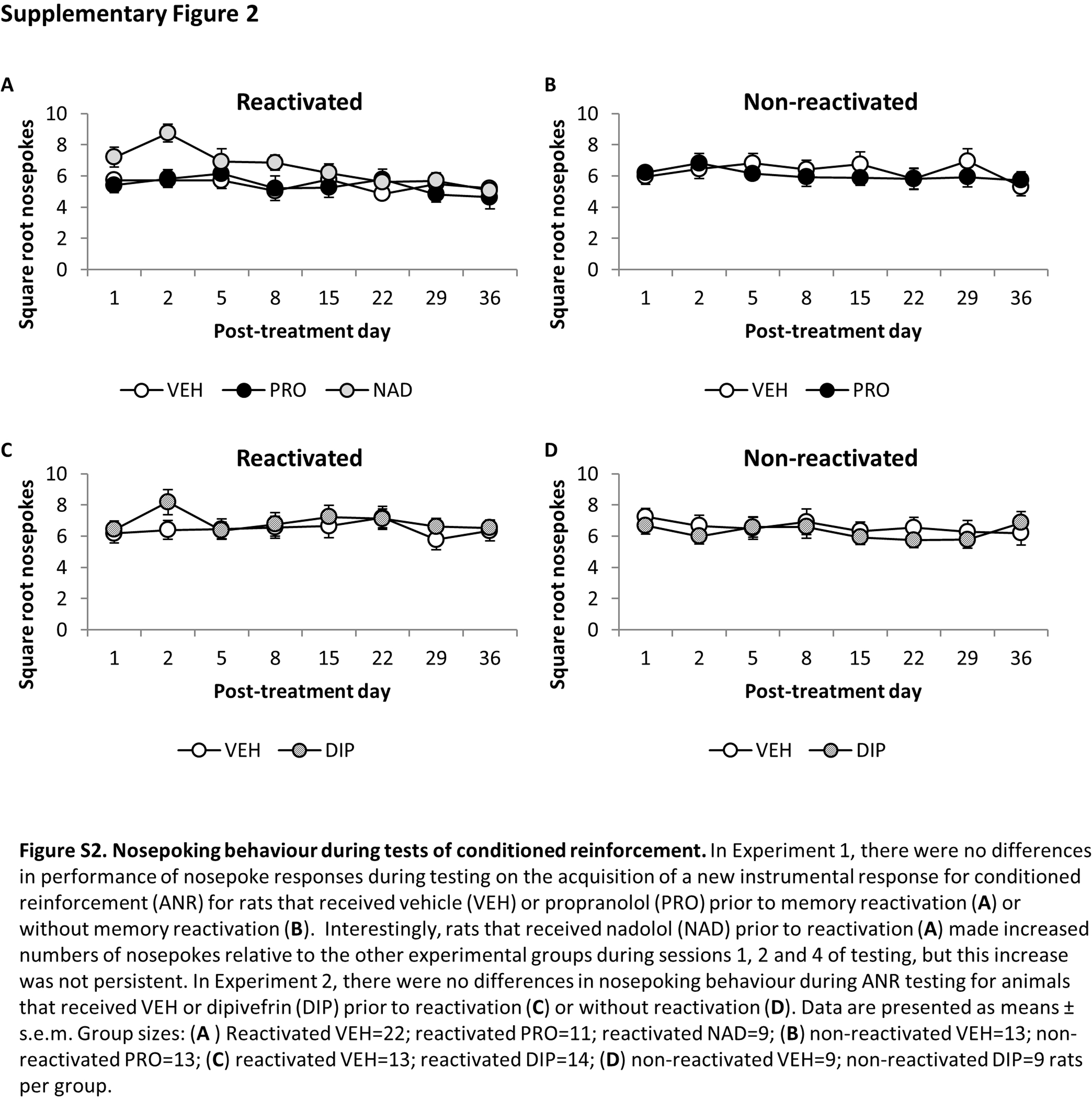

Supplement: Supplementary Figure S2 [file npp2015248x2.tif]

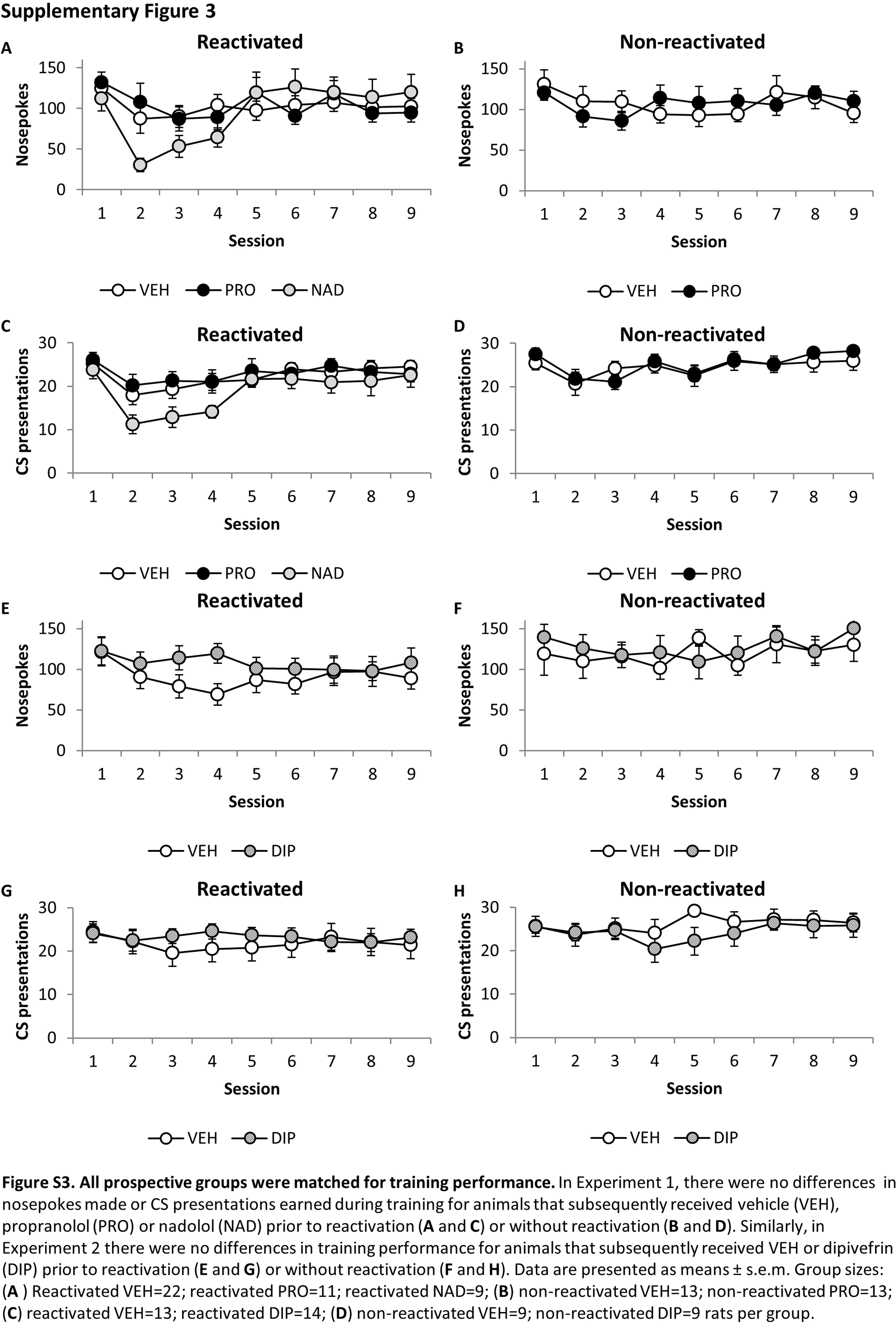

Supplement: Supplementary Figure S3 [file npp2015248x3.tif]
